# LncRNA FOXD2‐AS1 as a competitive endogenous RNA against miR‐150‐5p reverses resistance to sorafenib in hepatocellular carcinoma

**DOI:** 10.1111/jcmm.14465

**Published:** 2019-06-18

**Authors:** Chengjun Sui, Zhitao Dong, Cheng Yang, Minfeng Zhang, Binghua Dai, Li Geng, Jiongjiong Lu, Jiamei Yang, Minhui Xu

**Affiliations:** ^1^ Department of Special Treatment Ⅰ and Liver Transplantation Shanghai Eastern Hepatobiliary Surgery Hospital Shanghai China; ^2^ Department of Hepato‐Pancreato‐Biliary Surgery The Affiliated Suzhou Hospital of Nanjing Medical University Suzhou China

**Keywords:** hepatocellular carcinoma (HCC), long non‐coding RNA (lncRNA), pathogenesis, proliferation, resistance, sorafenib

## Abstract

The current study elucidated the role of a long non‐coding RNA (lncRNA), FOXD2‐AS1, in the pathogenesis of hepatocellular carcinoma (HCC) and the regulatory mechanism underlying FOXD2‐AS1/miR‐150‐5p/transmembrane protein 9 (TMEM9) signalling in HCC. Microarray analysis was used for preliminary screening of candidate lncRNAs in HCC tissues. qRT‐PCR and Western blot analyses were used to detect the expression of FOXD2‐AS1. Cell proliferation assays, luciferase assay and RNA immunoprecipitation were performed to examine the mechanism by which FOXD2‐AS1 mediates sorafenib resistance in HCC cells. FOXD2‐AS1 and TMEM9 were significantly decreased and miR‐150‐5p was increased in SR‐HepG2 and SR‐HUH7 cells compared with control parental cells. Overexpression of FOXD2‐AS1 increased TMEM9 expression and overcame the resistance of SR‐HepG2 and SR‐HUH7 cells. Conversely, knockdown of FOXD2‐AS1 decreased TMEM9 expression and increased the sensitivity of HepG2 and Huh7 cells to sorafenib. Our data also demonstrated that FOXD2‐AS1 functioned as a sponge for miR‐150‐5p to modulate TMEM9 expression. Taken together, our findings revealed that FOXD2‐AS1 is an important regulator of TMEM9 and contributed to sorafenib resistance. Thus, FOXD2‐AS1 may serve as a therapeutic target against sorafenib resistance in HCC.

## 1 INTRODUCTION

Hepatocellular carcinoma (HCC) is the fifth most prevalent cancer worldwide and the third most common cause of cancer‐related deaths.^1^, ^2^ HCC is an invasive malignant tumour that is generally diagnosed in an advanced stage, for which treatment is ineffective.^3^ Although significant progress has been achieved in the treatment of HCC, drug resistance and tumour recurrence still lead to a high mortality rate.^4^, ^5^ The clinical prognosis of HCC is extremely poor and the 5‐year survival rate is still quite low globally, mainly because of the high risk of vascular invasion, metastasis, drug resistance and recurrence after surgical resection.^6^ Therefore, unravelling the potential molecular mechanism underlying chemotherapeutic resistance in HCC (especially the changes of genetics and epigenetics) is a major focus of research activity.^7^

LncRNAs are RNA transcripts >200 nucleotides in length, but lack an obvious open reading frame.^8^, ^9^ Although lncRNA is not translated into protein, lncRNA participates in multiple physiological activities, including chromosome modification, transcription activation and interference, as well as cell growth, differentiation, and apoptosis.^10^ Recent studies have demonstrated that several abnormally expressed lncRNAs can mediate drug resistance. For instance, AFAP1‐AS1 has been reported to mediate cisplatin resistance in laryngeal cancer cells through the miR‐320a/RBP signalling pathway.^11^ Overexpression of lncRNA MALAT1 enhances autophagy and chemotherapeutic resistance of gastric cancer (GC) cells through the miR‐23B‐3P/ATG12 signalling pathway.^12^ The lncRNA, H19, up‐regulates expression of the multi‐drug resistance gene (MDR1), thereby promoting the accumulation of doxorubicin in HCC cells and increasing the acceptable level of toxicity 13; however, the role of the lncRNA, FOXD2‐AS1, in sorafenib‐resistant HCC cells remains elusive. In this study we determined the role of the lncRNA, FOXD2‐AS1, which is involved in resistance of HCC to sorafenib and elucidated the underlying mechanism.

## 2 MATERIALS AND METHODS

### 2.1 Patient specimens

In the current study, human HCC specimens were obtained from 60 patients who underwent surgery (34 males and 26 females) between January 2012 and September 2014 in the Department of Special Treatment Ⅰ and Liver Transplantation at Shanghai Eastern Hepatobiliary Surgery Hospital. No patient received radiotherapy or chemotherapy prior to tissue resection. HCC was diagnosed by three pathologists according to the WHO classification system. Tumour specimens were quickly frozen in liquid nitrogen after resection and immediately stored at −80°C. This study was approved by the Shanghai Eastern Hepatobiliary Surgery Hospital Ethics Committee and written informed consent was obtained from all patients before tissue acquisition.

### 2.2 Cell culture

HCC cell lines (HepG2 and HUH7) were purchased from the American Type Culture Collection (ATCC, Manassas, VA) and cultured in Dulbecco's modified Eagle's medium (DMEM) supplemented with 10% foetal bovine serum (FBS), and 100 U/mL of penicillin and 0.1 mg/mL of streptomycinat 37°C in 95% humidified air and 5% CO_2_. Sorafenib‐resistant HepG2 (SR‐HepG2) and ‐resistant HUH7 (SR‐HUH7) cells were prepared according to the method previously described.^2^

### 2.3 Cell viability

Cells were seeded into 96‐well plates and treated with different concentrations of sorafenib (catalognumber, S‐8502; LC Laboratories, Shanghai, China). Cell viability was examined by MTT assay. The half inhibitory concentration (IC_50_) value was determined for each HCC cell line. To evaluate the effect of lncRNA, cell viability was measured 96 hours after transfection using the MTT assay (Dojindo, Kumamoto, Japan) according to the manufacturer's instructions.

### 2.4 Microarray analysis

Microarray analysis of gene expression was performed according to the manufacturer's instructions (Agilent Technologies Co., Ltd., Santa Clara, CA). Briefly, 50 ng of purified mRNA was amplified and transcribed into double‐stranded complementary DNA (cDNA). As previously described,^8^ the cDNA was labelled and hybridized to human lncRNA Array v3.0 (Arraystar, Inc, Rockville, MD), according to the manufacturer's instructions. The original data were standardized and corrected using GenePix Pro 4.0 software. The comparison between HepG2 and SR‐HepG2 samples was analysed by a *t *test. LncRNAs with a *P* < 0.05 were selected and cluster analysis was carried out using the hierarchical method, average linkage and Euclidean distance metrics.

### 2.5 RNA isolation and qRT‐PCR

According to the manufacturer's instructions, total RNA was extracted from the cancer cells using TRIzol reagent (Invitrogen; Thermo Fisher Scientific, Inc, Waltham, MA). The first‐strand cDNA was synthesized using a PrimeScript 1st Strand cDNA synthesis kit (Takara Bio Inc, Kusatsu, Japan). The synthesized cDNA template was supplemented with SYBR Select Master Mix (Thermo Fisher Scientific). The following cycling conditions were used: pre‐denaturation at 95°C for 30 seconds; 35 denaturation cycles at 95°C for 5 seconds; annealing at 55°C for 40 seconds; extension at 72°C for 1 minute; and a final extension at 72°C for 10 minutes. qRT‐PCR was performed using the 7500 Real‐Time PCR system (Thermo Fisher Scientific). The level of lncRNA expression was normalized by the expression of GAPDH (△CT = target lncRNA Ct‐GAPDH Ct).

### 2.6 Western blot

Approximately 25 μg of protein was loaded onto gels for sodium dodecyl sulphate‐polyacrylamide gel electrophoresis (SDS‐PAGE), then transferred to nitrocellulose membranes (Bio‐Rad). The membranes were incubated with primary antibody (TMEM9, 1:1000;Cell Signaling Technology, Inc, Danvers, MA; Nrf2, 1:2000, Gene Tex, Irvine, CA; HO‐1, 1:800, Gene Tex; and GAPDH, 1:3000, Cell Signaling Technology), followed by the second antibody and visualized using an enhanced chemiluminescence kit (GE Healthcare, Chicago, IL).

### 2.7 Plasmid construction

The scramble shRNA sequence or shRNA targeting FOXD2‐AS1 (sh1 targets GCTTCCAGGTATGTGGGAA and sh2 targets GGACTCCACTCTTCGCTTA) was annealed and cloned into pGL3 vector (Promega, Madison, WI). Lentiviral particles expressing shRNA or FOXD2‐AS1 were produced in HEK293T cells, transfected into the cells for 48 hours, then the cells were treated with 1 mg/mL of puromycin (Promega Corporation, Madison, WI) for 4 days. To construct the luciferase reporter plasmids, we cloned the wild‐type (WT) FOXD2‐AS1 with the potential miR‐150‐5P binding site or mutant of this site into the downstream luciferase gene in the pmirGL3 reporter vector. Similarly, the predicted binding sites of miR‐150‐5P (WT and mutant) in TMEM9 3’‐UTR were cloned into the pmirGL3 reporter vector. These plasmids were designated as FOXD2‐AS1, FOXD2‐AS1‐mut, TMEM9 3’UTR and TMEM9 3’UTRmut, respectively. TMEM9 full‐length cDNA was amplified and cloned into the pCMV‐C‐Flag vector (Beyotime, Shanghai, China) with FLAG‐tag at the C‐terminus.

### 2.8 Dual‐luciferase reporter gene assay

Dual‐luciferase reporter gene assay was carried out, as described below. Cells (3 × 10^5^) were cultured in 24‐well plates and cotransfected with 2 ng pRL‐TK (Promega) 10 ng of luciferase plasmids, and 100 ng of miR‐150‐5p mimic or negative control. The luciferase activity in the cells was detected 48 hours after transfection using a luciferase assay kit (Promega) and standardized with Ranilla luciferase activity. The experiments were repeated three times.

### 2.9 Cell transfection

The chemically synthesized TMEM9‐specific siRNA (5′‐GAATGACACAGCAATGAA‐3′) miR‐150‐5p mimics, miR‐150‐5p inhibitors and miRNA controls (miR‐NC) were purchased from GENECHEM (Shanghai, China). Using Lipofectamine 3000 (Invitrogen), TMEM9‐FLAG plasmid, TMEM9 siRNA, scrambled siRNA, miRNA mimics, miRNA inhibitors and miRNA controls were transfected into the cells according to the manufacturer's instructions.

### 2.10 Statistical analysis

All statistical analyses were performed using GraphPad Prism 6.0 software (GraphPad Software, Inc, La Jolla, CA). The thrice‐repeated data are expressed as the mean ± standard deviation (SD). Inter‐group comparisons were performed using *t* tests or one‐way ANOVA. The correlation between the FOXD2‐AS1 level and TMEM9 or miR‐150‐5p level was analysed with the Pearson correlation coefficient. A *P* < 0.05 was considered statistically significant.

## 3 RESULTS

### 3.1 Down‐regulated expression of FOXD2‐AS1 in sorafenib‐resistant HCC cells

**Figure 1:**
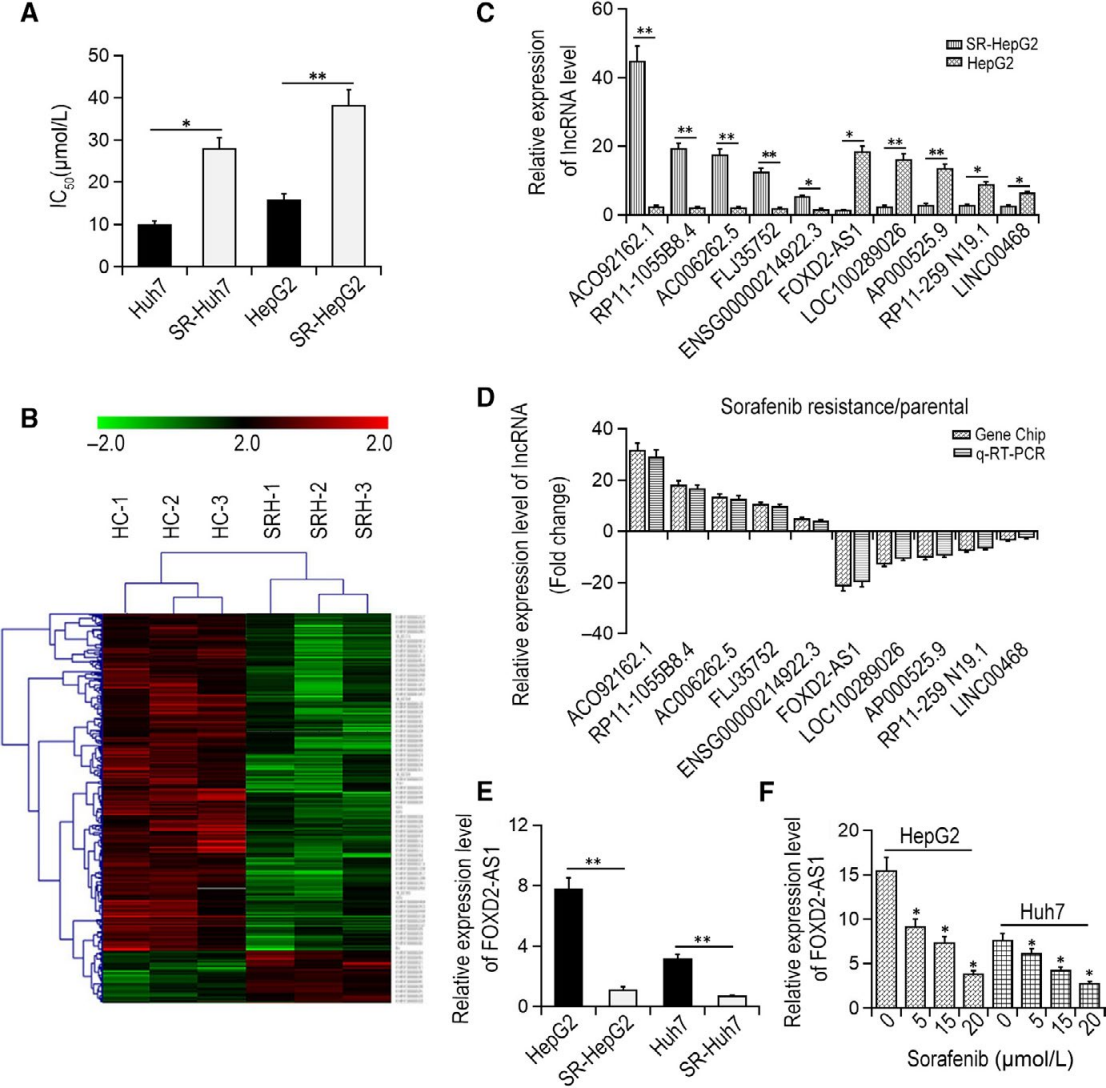
Down‐regulation of FOXD2‐AS1 was correlated with sorafenib resistance in hepatocellular carcinoma (HCC) cells. (A) IC_50_ values of sorafenib in HCC cells. *P < 0.05, **P < 0.01. (B) Differential expression levels of lncRNAs between SR‐HepG2 and SR‐HUH7 cells were detected by microarray analysis. (C) qRT‐PCR verified 10 differentially expressed lncRNAs in SR‐HepG2 and SR‐HUH7 cells. *P < 0.05, **P < 0.01. (D) The expression profile of differentially expressed lncRNAs was compared by microarray gene chip and qRT‐PCR. (E) The mRNA expression levels of FOXD2‐AS1 in HCC cells. **P < 0.01. (F) qRT‐PCR was performed to detect the expression of FOXD2‐AS1 mRNA in HepG2 and Huh7 cells treated with sorafenib at different doses. *P < 0.05 vs the 0 μmol/L group

To clarify the relationship between lncRNAs and sorafenib resistance in HCC cells, sorafenib‐resistant cell lines (SR‐HepG2 and SR‐HUH7) were constructed according to an established protocol. As illustrated in Figure 1A, the half maximal inhibitory concentration (IC_50_) value ranged from 9.8 μmol/L in Huh7 cells to 31.7 μmol/L in SR‐HepG2 cells. Cells that exhibited higher IC50 values were defined as resistant. Using a lncRNA microarray assay, we analysed aberrantly‐expressed lncRNAs between SR‐HepG2 and HepG2 cells. Compared with the HepG2 cells, 3016 lncRNAs were differentially expressed in SR‐HepG2 cells, including 1803 up‐regulated lncRNAs and 1213 down‐regulated lncRNAs (fold change ≥ 2.0, *P* < 0.05, Figure 1B). We then selected the lncRNA with the largest differential expression for subsequent qRT‐PCR validation (Figure 1C,D). Among the differentially expressed lncRNAs, FOXD2‐AS1 was the most down‐regulated lncRNA in SR‐HepG2 cells compared with the parent HepG2 cells (data not shown). Expression of FOXD2‐AS1 in SR‐HepG2 and SR‐HuH7 cells was changed by −17.6‐ and −7.5‐fold compared with the parent cells (*P* < 0.01, Figure 1E). Moreover, sorafenib down‐regulated the level of FOXD2‐AS1 expression in HepG2 and HUH7 cells in a dose‐dependent manner (Figure 1C). Taken together, these results suggest that FOXD2‐AS1 plays a key role in sorafenib resistance in HCC.

### 3.2 FOXD2‐AS1 binds to miR‐150‐5p

**Figure 2:**
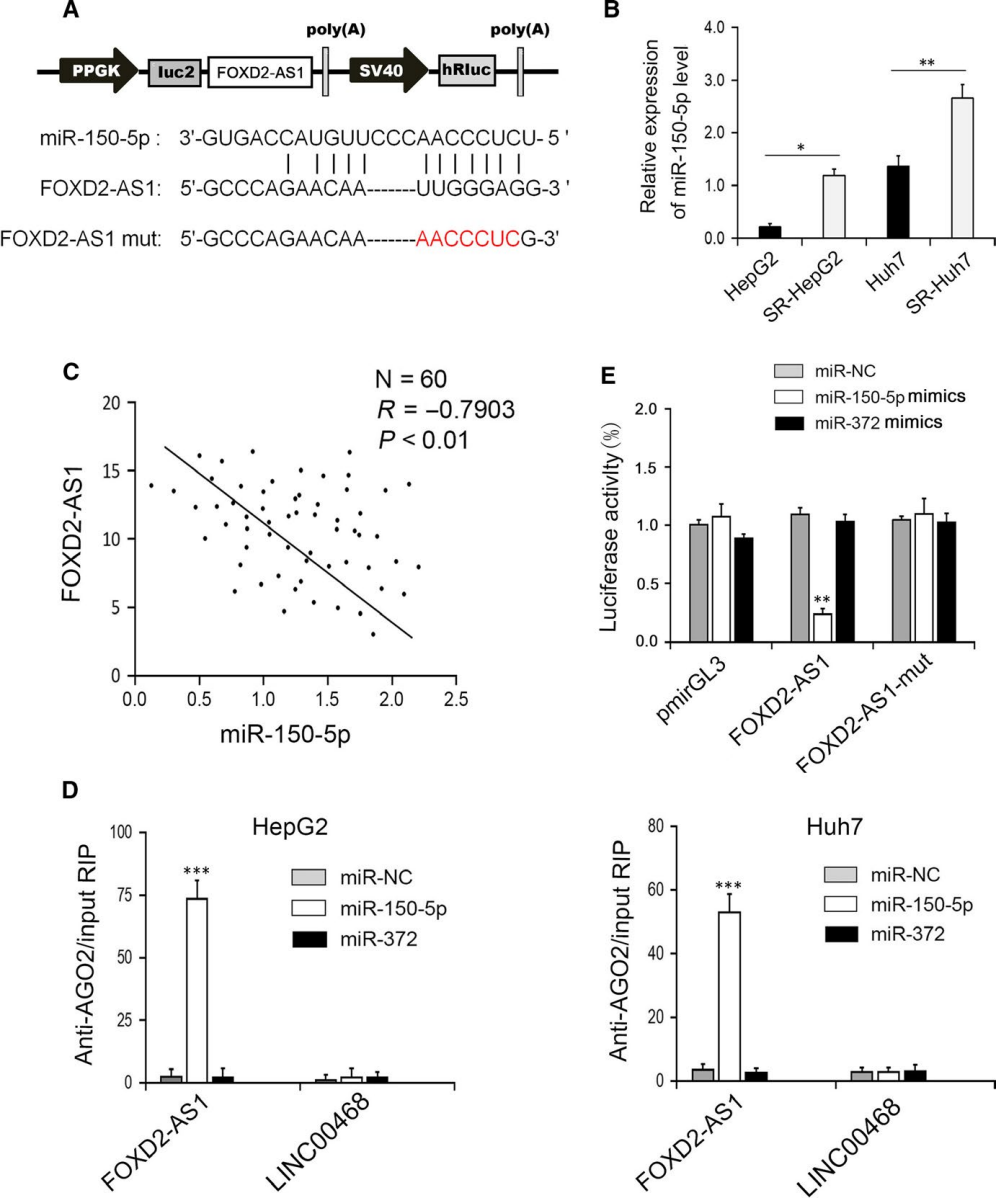
FOXD2‐AS1 directly acts on miR‐150‐5p. (A) Schematic diagram of miR‐150‐5p binding sites in FOXD2‐AS1. (B) qRT‐PCR showed the relative expression of miR‐150‐5p in SR‐HepG2 and SR‐HUH7 cells compared with that in respective parent cells. *P < 0.05，**P < 0.01. (C) Correlation analysis between miR‐150‐5p and FOXD2‐AS1 expression in hepatocellular carcinoma samples (*r* = −0.7903, *P* < 0.01). (D) Anti‐AGO2 RIP was performed in HepG2 and HUH7 cells, ***P < 0.001 vs miR‐NC. (E) Luciferase activity in HEK293T cells cotransfected with miR‐150‐5p and pmirGL3, FOXD2‐AS1 or FOXD2‐AS1‐mut. **P < 0.01 vs miR‐NC

To determine whether FOXD2‐AS1 binds miRNAs, the potential binding force of miRNAs and FOXD2‐AS1 was predicted using starBase v.2.0 software (Figure 2A). Among the potential miRNAs, the expression of miR‐150‐5p was significantly up‐regulated in sorafenib‐resistant HCC cells (Figure 2B). In HCC tissue specimens, FOXD2‐AS1 was negatively correlated with miR‐150‐5p expression (Figure 2C). Moreover, endogenous FOXD2‐AS1 precipitated by AGO2 tended to be enriched in cells overexpressing miR‐150‐5p, but not miR‐372 (Figure 2D). Additionally, the dual‐luciferase assay showed that cotransfection of miR‐150‐5p mimics with FOXD2‐AS1 WT (pmirGLEWT‐FOXD2‐AS1), rather than the pmirGLE‐mut‐FOXD2‐AS1 mutant, significantly reduced luciferase activity (Figure 2E). Together, these results suggest that FOXD2‐AS1 binds to miR‐150‐5p.

### 3.3 FOXD2‐AS1 enhances TMEM9 expression

**Figure 3:**
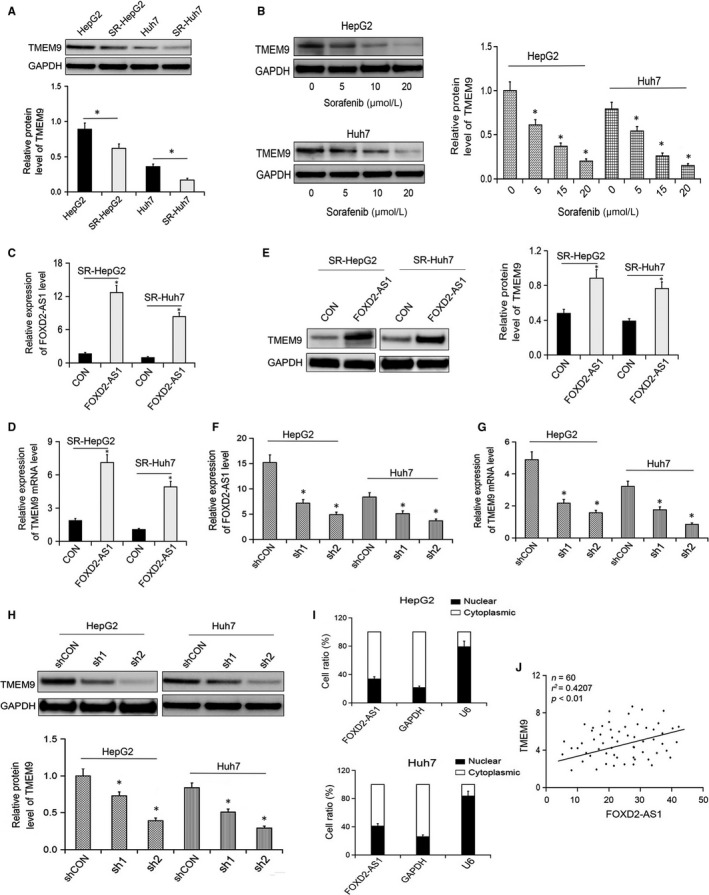
FOXD2‐AS1 regulates TMEM9 expression. (A) TMEM9 expression in HepG2, HUH7, RS‐HepG2 and RS‐HUH7 cells. (B) TMEM9 in HepG2 and HUH7 cells treated with sorafenib. (C) FOXD2‐AS1 expression in SR‐HepG2 and SR‐HUH7 cells 48 h after transfection with lentivirus expressing FOXD2‐AS1 or empty vector. (D,E) TMEM9 expression in SR‐HepG2 and SR‐HUH7 cells overexpressing FOXD2‐AS1. (F) FOXD2‐AS1 expression in HepG2 and Huh7 cells 48 h after shRNA1 or 2. (G‐H) The expressions of TMEM9 in SR‐HepG2 and SR‐HUH7 cells overexpressing FOXD2‐AS1. (I) FOXD2‐AS1 was mainly distributed in the cell cytoplasm. (J) Pearson correlation analysis was conducted to evaluate the correlation between fox FOXD2‐AS1 and TMEM9 mRNA in hepatocellular carcinoma tissue samples. *P < 0.05

TMEM9 is an important regulator in the progression of HCC.^14^ Interestingly, we found that the expression of TMEM9 was significantly lower in SR‐HepG2 and SR‐HUH7 cells than HepG2 and HUH7 cells (Figure 3A). Moreover, sorafenib reduced the expression of TMEM9 in a dose‐dependent manner (Figure 3B). Stable overexpression of FOXD2‐AS1 in SR‐HepG22 and SR‐HUH7 cells significantly up‐regulates the expression of TMEM9 at the mRNA and protein levels (Figure 3C‐E). Conversely, silencing of FOXD2‐AS1 in HepG2 and HUH7 cells significantly down‐regulated the expression of TMEM9 (Figure 3F‐H). Cell fractionation testing revealed that FOXD2‐AS1 was mainly located in the cytoplasm of HCC cells, implying that FOXD2‐AS1 might play a role in post‐transcriptional modification (Figure 3I). Moreover, the expression of FOXD2‐AS1 in HCC tissue samples was positively correlated with TMEM9 expression (*R* ^2^ = 0.4207, *P* < 0.05, Figure 3J). Collectively, these results suggest that TMEM9 is a target of FOXD2‐AS1 in HCC.

### 3.4 FOXD2‐AS1 regulates TMEM9 expression by acting as aceRNA of miR‐150‐5p

**Figure 4:**
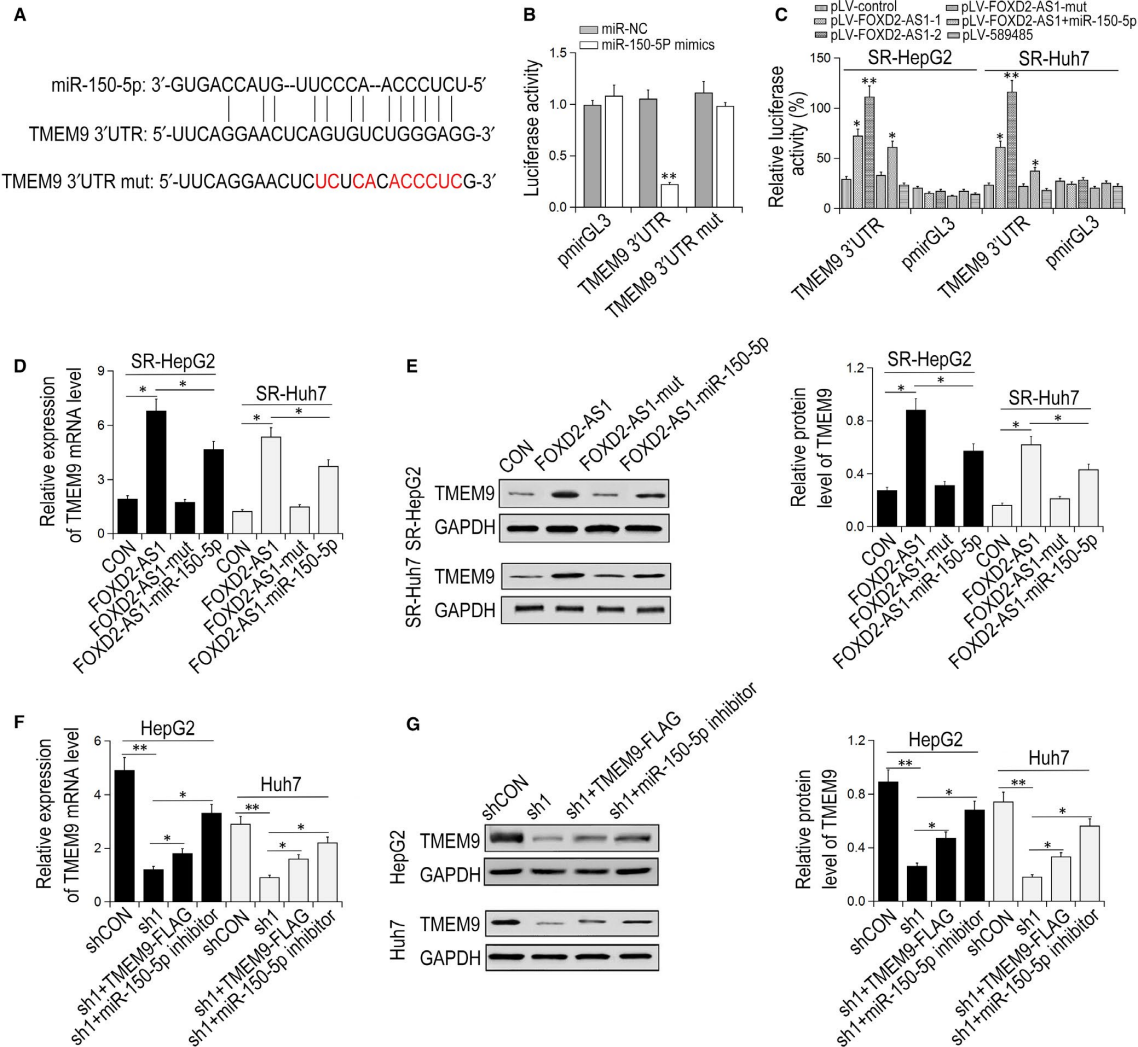
FOXD2‐AS1 targets TMEM9 by acting as a ceRNAof miR‐150‐5p. (A) Schematic diagram of miR‐150‐5p binding sites in 3′‐UTR of TEEM9. (B) Luciferase activity in HEK293T cells, **P < 0.01 vs miR‐NC. (C) Plasmids overexpressing WT or Mut FOXD2‐AS1 and luciferase reporter vector containing TMEM9 3′‐UTR or empty vector were cotransfect into SR‐HepG2 and SR‐HUH7 cells. (D‐E) The expression of TMEM9in SR‐HEPG2 and SR‐HUH7 cells transfected with FOXD2‐AS1 or FOXD2‐AS1‐mut plasmids with or without miR‐150‐5p mimics. (F‐G) qRT‐PCR and Western blot detected the expression of TMEM 9 in HepG2 and Huh7 cells transfected with FOXD2‐AS1shRNA1 with or without miR‐150‐5p inhibitor. *P < 0.05. **P < 0.01

The luciferase reporter assay showed that transfection with miR‐150‐5p mimics significantly reduced the activity of TMEM9 3’UTR, but had no apparent effect on TMEM9 3’UTR mut (Figure 4A,B). Overexpression of FOXD2‐AS1, but not FOXD2‐AS1‐mut, remarkably increased the luciferase activity of TMRM9 3′UTR, whereas cotransfection with miR‐150‐5p inhibited this effect (Figure 4C). Overexpression of FOXD2‐AS1, but not FOXD2‐AS1‐mut, significantly up‐regulated TMEM9 expression and this effect was restrained by cotransfection with miR‐150‐5p mimics (Figure 4D,E). Conversely, FOXD2‐AS1 knockdown down‐regulated TMEM9 expression and overexpression of TMEM9 or treatment with miR‐150‐5p inhibitor partially restored TMEM9 expression reduced by FOXD2‐AS1 knockdown (Figure 4F,G). Collectively, these results suggest that FOXD2‐AS1 modulates TMEM9 expression through competitive binding with miR‐150‐5p.

### 3.5 FOXD2‐AS1 reverses sorafenib resistance in HCC cells by regulating TMEM9 expression

**Figure 5:**
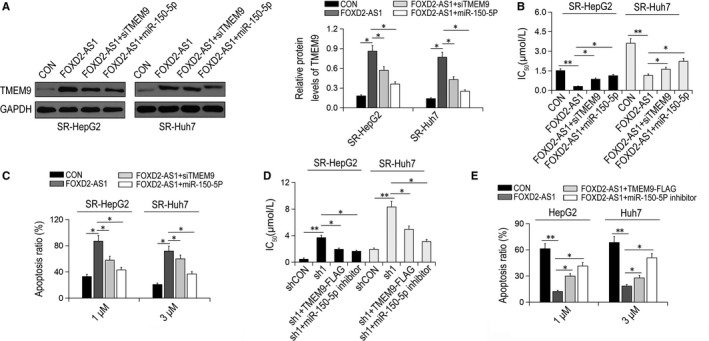
FOXD2‐AS1 reverses sorafenib resistance by miR‐150‐5p/TMEM9 axis. (A) TMEM9 in FOXD2‐AS1 overexpressed SR‐HepG2 and SR‐HUH7 cells transfected with TMEM9 siRNA or miR‐150‐5p mimics. (B) IC50 of sorafenib in FOXD2‐AS1‐overexpressing SR‐HepG2 and SR‐HUH7 cells transfected with TMEM9 siRNA or miR‐150‐5p mimics. (C) Apoptosis rates of transfected SR‐HepG2 and SR‐HUH7 cells after treatment with sorafenib for 48 h. *P < 0.05. (D) IC50 of sorafenib in HepG2 and HUH7 cells with FOXD2‐AS1 knockdown transfected with TMEM9‐FLAG plasmid or miR‐150‐5p inhibitor. (E) Apoptosis rates of transfected HepG2 and Huh7 cells after sorafenib treatment. *P < 0.05, **P < 0.01

We then determined whether FOXD2‐AS1 regulates sorafenib resistance via the miR‐150‐5p/TMEM9 axis. Compared with control cells, overexpression of FOXD2‐AS1 significantly increased the sensitivity of SR‐HepG2 and SR‐Huh7 cells to sorafenib resistance, whereas knockdown of TMEM9 or overexpression of miR‐150‐5p reversed this effect (Figure 5A,B). Similarly, FOXD2‐AS1 increased the apoptotic rates of SR‐HepG2 and SR‐Huh7 cells and TMEM9 depletion or miR‐150‐5p overexpression restrained the apoptosis induced by FOXD2‐AS1 (Figure 5C). Conversely, knockdown of FOXD2‐AS1 increased sorafenib resistance and reduced apoptosis in SR‐HepG2 and SR‐Huh7 cells, whereas overexpression of TMEM9 or treatment with miR‐150‐5p inhibitor reversed the effects mediated by FOXD2‐AS1 depletion (Figure 5D,E). Together, these results suggest that FOXD2‐AS1 contributes to sorafenib resistance by targeting the miR‐150‐5p‐5p/TMEM9 axis.

### 3.6 FOXD2‐AS1 inhibits the NRf2 signalling pathway by regulating TMEM9 expression

**Figure 6:**
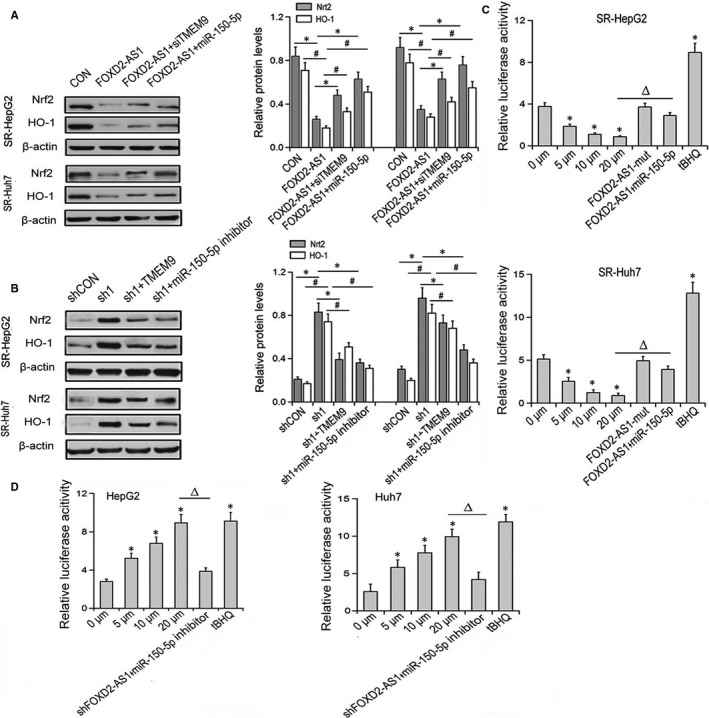
FOXD2‐AS1 regulates Nrf2 signalling pathway. (A) Nrf2 and HO‐1 in FOXD2‐AS1‐overexpressing cells transfected with TMEM9 siRNA or miR‐150‐5p mimics. *P < 0.05 vs Con for Nrf2 comparison; ^#^ *P* < 0.05 vs Con for HO‐1 comparison. (B) The expression levels of Nrf2 and HO‐1 in FOXD2‐AS1‐silencing cells transfected with TMEM9‐FLAG plasmid or miR‐150‐5p inhibitor. *P < 0.05 vs Con for Nrf2 comparison; ^#^ *P* < 0.05 vs Con for HO‐1 comparison. (C) In the presence or absence of miR‐150‐5p mimics, FOXD2‐AS1 or FOXD2‐AS1‐mut plasmids were transfected into SR‐HepG2 and SR‐Huh7 cells. (D) In the presence or absence of miR‐150‐5p inhibitor, shRNA‐FOXD2‐AS1 or FOXD2‐AS1‐mut plasmids were transiently transfected into HEPG2 and HUH7 cells; *P < 0.05 vs the 0 μmol/L group, ^Δ^P<0.05 compared with the 20 μmol/L group

Western blot analysis demonstrated FOXD2‐AS1 overexpression decreased the protein levels of Nrf2 and HO‐1 levels in SR‐HEPG2 and SR‐HUH7 cells, whereas silencing of TMEM9 or ectopic expression of miR‐150‐5p partially restored the expression of Nrf2 and HO‐1 (Figure 6A). Conversely, FOXD2‐AS1 depletion increased the levels of Nrf2 and HO‐1 in HEPG2 and HUH7 cells, whereas ectopic expression of TMEM9 or silencing of miR‐150‐5p partially reversed these effects (Figure 6B). Moreover, in SR‐HepG2 and SR‐HUH7 cells, the ARE‐driven luciferase activity was decreased by FOXD2‐AS1 in a dose‐dependent manner, which was partially reversed by overexpression of miR‐150‐5p (Figure 6C). In HepG2 and HUH7 cells with the silencing of FOXD2‐AS1, the ARE‐driven luciferase activity was increased in a dose‐dependent pattern, and this effect was partially blocked by miR‐150‐5p inhibitor (Figure 6D).

## 4 DISCUSSION

Sorafenib is regarded as a standard chemotherapy for advanced HCC in clinical trials; however, the low clinical efficacy limits the use of sorafenib.^15^, ^16^, ^17^, ^18^, ^19^ Although biotechnological progress has been achieved in the past few decades, the precise molecular mechanism underlying sorafenib resistance has not been fully unravelled. In this study we demonstrated downregulation of FOXD2‐AS and increased TMEM9 expression in HepG2 and HUH7 cells with sorafenib resistance.

Recent studies have confirmed that lncRNAs play an important functional role in multidrug resistance of cancer cells. Specifically, lncRNA AK126698 is involved in cisplatin resistance in non‐small cell lung cancer cells and overexpression of lncRNA snaR enhances sorafenib‐induced cell death in colon cancer.^20^ It has been reported that the lncRNA, LEIGC, mediates sorafenib resistance and epithelial‐mesenchymal transition in gastric cancer.^21^ Linc‐TUG1 provokes impaired sensitivity in oesophageal squamous cell carcinoma.^22^ FOXD2‐AS1 knockdown inhibits the tumour growth of gemcitabine‐resistant bladder cancer cells via the miR‐143/ABCC3 axis.^23^ In the present study, a group of lncRNAs differentially expressed in sorafenib‐resistant HCC cells were validated.

In this study, we confirmed that down‐regulation of FOXD2‐AS1 and TMEM9 expression was positively correlated with the increase in sorafenib resistance. Further investigation demonstrated that FOXD2‐AS1 regulated TMEM9 expression by completely sponging miR‐150‐5p, which inhibited miR‐150‐5p‐mediated degradation of TMEM9 mRNA. Indeed, this is the first study to confirm that FOXD2‐AS1 regulates TMEM9 expression by acting as a ceRNA ofmiR‐150‐5p. Ectopic expression of FOXD2‐AS1 reversed sorafenib resistance in HCC cells, whereas silencing of TMEM9 or overexpressing mir‐150‐5p partially restored this effect, indicating that FOXD2‐AS1 regulates sorafenib resistance via miR‐150‐5p/TMEM9 axis. In addition, the dual‐luciferase assay confirmed that FOXD2‐AS1 increased TMEM9 expression and suppressed the Nrf2 signalling pathway in SR‐HepG2 and SR‐HUH 7 cells, and these effects were partially blocked by miR‐150‐5p mimics. In contrast, inHepG2 and HUH7 cells with silencing of FOXD2‐AS1, ARE‐driven luciferase activity was increased in a dose‐dependent manner, which was partially blocked by miR‐150‐5p inhibitor. These results indicate that FOXD2‐AS1 regulates the Nrf2 signalling pathway via the miR‐150‐5p/TMEM9 pathway.

## 5 CONCLUSION

Taken together, FOXD2‐AS1 is a novel key regulator of TMEM9 and mediates sorafenib resistance in HCC cells. FOXD2‐AS1 competes with the 3'UTR of TMEM9 for binding with miR‐150‐5p, which promotes the expression of TMEM9, inhibits the Nrf2‐ARE signalling pathway, and reverses sorafenib resistance in HCC cells. The finding that the FOXD2‐AS1/miR‐150‐5p/TMEM9 signalling pathway is involved in sorafenib resistance may provide novel strategies to overcome sorafenib resistance in HCC.

## CONFLICT OF INTERESTS

The authors declare that they have no competing interests.

## Data Availability

Data are available in this manuscript.
